# The evolution of medulloblastoma therapy to personalized medicine

**DOI:** 10.12688/f1000research.10859.1

**Published:** 2017-04-13

**Authors:** Soma Sengupta, Daniel Pomeranz Krummel, Scott Pomeroy

**Affiliations:** 1 Winship Cancer Institute, Emory University Hospital, Atlanta, GA, USA; 2 F.M. Kirby Neurobiology Center, Boston Children’s Hospital, Boston, MA, USA

**Keywords:** Medulloblastoma, specific targeted therapeutics, WNT, SHH, TP53

## Abstract

Recent advances in cancer genomics have revolutionized the characterization and classification of medulloblastomas. According to the current WHO guidelines, medulloblastomas are now classified into the following molecularly defined groups: Wnt signaling pathway (WNT)-activated, sonic hedgehog signaling pathway (SHH)-activated and tumor suppressor protein p53 (TP53)-mutant, SHH-activated and TP53-wildtype, and non-WNT/non-SHH (i.e. group 3 and group 4). Importantly, genomic, epigenomic, and proteomic advances have created a potential paradigm shift in therapeutic options. The challenge now is to (i) translate these observations into new therapeutic approaches and (ii) employ these observations in clinical practice, utilizing the classification following a molecular analysis for diagnosis and application of new subgroup-specific targeted therapeutics.

## Introduction

Medulloblastomas account for 12% of childhood brain tumors ^1^. Approximately 80% of medulloblastomas occur in children under the age of 15. In adults, medulloblastomas are rare (1–2%). To date, the 5-year survival rate for children with average and high-risk disease as defined by clinical criteria is 80% and 60–65%, respectively ^2^. Recent advances in cancer genomics have led to a fundamental change in medulloblastoma classification. Based on genome-wide transcription profiling, it has been shown that medulloblastomas comprise at least four molecular subgroups ( Table 1), each with unique transcription profiles, mechanism of tumorigenesis, and clinical outcome ^3– 6^. Each of these subgroups, Wnt signaling pathway (WNT) (10% of medulloblastomas), sonic hedgehog signaling pathway (SHH) (30%), group 3 (15%), and group 4 (45%), will be discussed in the following sections.

**Table 1.** Features of medulloblastoma subgroups.

Subtype	Molecular characteristics	Mutations	Age group
**WNT activated**	WNT pathway activation	CTNNB1 DDX3X Chromatin-remodeling genes TP53	Least common of subgroups Found in children and adults, not infants
**SHH activated and TP53 wild-** **type**	SHH pathway activation	PTCH1 SMO SUFU TERT promoter Chromatin-remodeling genes	Infants, children, and adults
**SHH activated and TP53** **mutant**	SHH pathway activation	TP53	5–18 years old
**Group 3**	Elevated expression of MYC GABRA5 over-expression	SMARCA4 Chromatin-remodeling genes Genes of TGF-β pathway	Infants and children, not adults More common in boys than in girls
**Group 4**	Lmx1A expression	Chromatin-remodeling genes	More common in children than in adults Least common in infants

CTNNB1, catenin beta 1; DDX3X, DEAD-box helicase 3; Lmx1a, LIM homeobox transcription factor; PTCH1, Patched-1; SHH, sonic hedgehog; SMARCA4, SWI (switching)/SNF (sucrose non-fermenting)-related, matrix-associated, actin-dependent regulator of chromatin, subfamily A, member 4; SMO, smoothened receptor; SUFU, suppressor of fused homolog protein; TERT, telomerase reverse transcriptase; TGF-β, transforming growth factor beta; TP53, tumor suppressor protein p53.

## Clinical behavior

**Figure 1.**
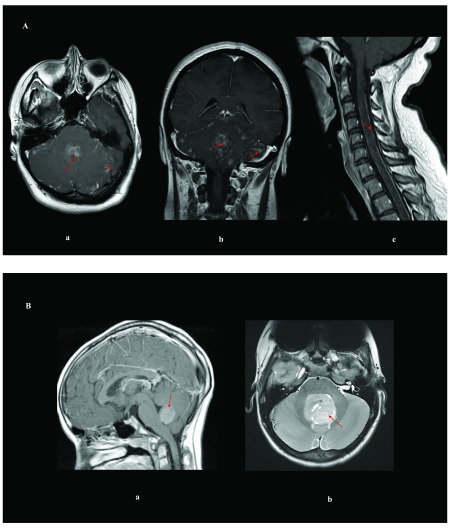
Imaging of pediatric and adult medulloblastomas. ( **A**) Magnetic resonance imaging of an adult woman who has medulloblastoma in the brain and spine with leptomeningeal spread: ( **a**) axial T1 of the brain post-gadolinium contrast; ( **b**) coronal T1 of the brain post-gadolinium contrast; ( **c**) sagittal T1 of the cervical spine post-gadolinium contrast. ( **B**) Brain magnetic resonance imaging of pediatric medulloblastomas: ( **a**) sagittal post-gadolinium WNT tumor; ( **b**) axial T2 of a SHH tumor. Red arrows delineate the tumor/leptomeningeal disease.

Medulloblastomas typically occur in the cerebellum and are primarily a pediatric brain cancer. Historically, medulloblastomas were described as “small round blue cell tumors” ^7^. The tumors are highly cellular with minimal cellular differentiation and have been defined by histological features that do not accurately predict clinical outcome (classic, desmoplastic/nodular, large-cell/anaplastic, medulloblastoma with neuroblastic features, medulloblastoma with glial differentiation, medullomyoblastoma, and melanotic medulloblastoma). Approximately one-third of the tumors have metastatic spread through CSF pathways, which puts the tumor into a “high-risk” group and is associated with poor outcome. In terms of MRI imaging characteristics, an adult medulloblastoma patient exhibits disseminated leptomeningeal disease in the brain and spinal cord ( Figure 1A), while pediatric medulloblastoma patients of the WNT subgroup tend to be in the cerebellar peduncle, patients of the SHH subgroup tend to be in the cerebellar hemisphere, but can be centrally located, as shown in Figure 1B, and midline cerebellar tumors tend to belong to groups 3 and 4 or SHH ^8^.

## Treatment: current standard of care

Medulloblastomas are clinically categorized as average-risk and high-risk disease ^9^ ( Table 2). Maximal surgical resection, in tumors that are amenable to surgery, is the first step in all cases. However, cerebellar mutism (severely diminished or absent speech output) can be an acute post-surgical complication in up to one-quarter of patients, which usually partially recovers, although survivors typically are left with dysarthria and neurocognitive issues ^10^. For average-risk disease, patients receive craniospinal radiotherapy (23.4 Gy in 30 fractions, followed by conformal tumor bed boost to 54–56 Gy over 6 weeks) with or without vincristine. After the radiation, children older than 3 years with non-disseminated medulloblastoma receive eight cycles of vincristine (mitotic inhibitor), cisplatin (DNA cross-linker), and two alkylating agents – cyclophosphamide and CCNU (lomustine) chemotherapy – for approximately 1 year. For poor-risk disease, craniospinal radiotherapy is given at a higher dose (36–39.6 Gy in 30 fractions, followed by posterior fossa boost to 54–56 Gy over 6 weeks) and chemotherapy (agents used include cisplatin, cyclophosphamide, and vincristine) ^9, 11^. Sometimes, stem cell transplants are also offered prior to the initiation of therapy. The craniospinal radiation and chemotherapy regimens described are also for the most part used in adult patients, but the use of post-adjuvant chemotherapy has not been shown to improve survival. In addition, vincristine and cisplatin cause significant toxicity. Infants younger than 3 years are often treated with high-dose chemotherapy and stem cell rescue regimens to delay the time to or avoid completely the administration of craniospinal irradiation ^9, 11^.

Unfortunately, these treatment regimens come with considerable morbidity. For example, the majority of children treated with intensive chemotherapy and irradiation (especially infants and those exposed to higher doses) are at risk for significant hearing loss, endocrine and neurocognitive deficits, and secondary benign and malignant tumors ^11^.

**Table 2.** Staging and risk stratification of medulloblastomas.

Modified Chang Staging			
T stage		M stage	
T1	Tumor <3 cm in diameter	M0	No evidence of gross subarachnoid or hematogenous metastasis
T2	Tumor ≥3 cm in diameter	M1	Microscopic tumor cells found in CSF
T3a	Tumor >3 cm and with extension into aqueduct of Sylvius or foramen of Luschka	M2	Gross nodular seeding intracranially beyond the primary site (in cerebellar/cerebral subarachnoid space or in third or lateral ventricle)
T3b	Tumor >3 cm and with unequivocal extension into brainstem	M3	Gross nodular seeding in spinal subarachnoid space
T4	Tumor >3 cm with extension past aqueduct of Sylvius or down past foramen magnum	M4	Metastasis outside cerebrospinal axis
Risk Stratification			
Standard (Average) Risk (66%)		High Risk (34%)	
>3 years old		<3 years old	
<1.5 cm ^2^ residual disease after resection		Subtotal resection, >1.5 cm ^2^ residual tumor	
M0 by craniospinal MRI and CSF		M+, leptomeningeal seeding, and location outside of the posterior fossa	

CSF, cerebrospinal fluid; MRI, magnetic resonance imaging.

## Potentially new treatments and approaches

With the advent of genomics, it has become increasingly clear that medulloblastoma is not a discrete entity, as shown by the recent WHO classification ^8^. Currently, there is a planned clinical study looking at the feasibility of surgery and chemotherapy in children with Wnt-positive medulloblastoma (NCT02212574). A Pediatric Brain Tumor Consortium study evaluated the use of GDC-0449 (vismodegib, Genentech Corporation, USA), which blocks a key protein (Smoothened, or SMO) in the SHH signaling pathway in medulloblastoma, and, as anticipated, patients with the SHH subtype who had the *SMO/PTCH* mutation responded to this drug ^12^. However, even in this group who responded to the vismodegib, it was transient with resistance developing quickly. Vismodegib has had some success in the recurrent SHH subgroup setting ^12, 13^. A proposed consensus for the design of next-generation clinical trials was discussed by Ramaswamy *et al.* ^14^ and is summarized in Table 3.

**Table 3.** Adapted table of a proposed consensus for designing the next generation of clinical trials in medulloblastoma (Ramaswamy *et al.* ^14^).

**Medulloblastoma patient subgroups → genome-wide** **methylation array → molecularly informed clinical trial or** **other validated methods**
**Tissue collection from all patients → snap-frozen and** **paraffin-embedded tumor tissue, blood, and CSF**
**All patients require tumor board planning for a clinical trial** **registry: neuroimaging, neuropathology, and radiotherapy**
**Treatment-related side effects in all patients in the short and** **long term: quality-of-life measures and neuropsychological** **outcomes**
**Recurrent disease: tumors should be re-biopsied if the** **diagnosis was unclear, or 2 years after the initial diagnosis,** **or before using targeted therapy for 2 years**
**Extent of resection: neurosurgeons should aim for maximal** **safe removal**

CSF, cerebrospinal fluid.

## WNT subgroup of medulloblastoma

**Figure 2.**
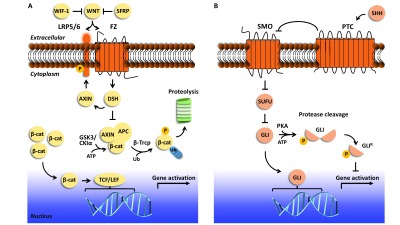
WNT and SHH signaling pathways. ( **A**) The WNT signaling pathway is mediated by the receptor Frizzled (FZ) and single-pass low-density lipoprotein receptor-related protein 5 or 6 (LRP5/6). In the pathway’s “off” state (in the event of low, no, or WNT ligand function inhibited by WNT inhibitor factor 1 [WIF-1] or secreted frizzle-related protein [SFRP]), β-catenin (β-cat) is targeted for phosphorylation by glycogen synthase kinase 3 (GSK3) and casein kinase I alpha (CKIα), aided by proteins AXIN and adenomatous polyposis coli (APC). β-catenin is then ubiquitinated and targeted for proteolysis by the proteasome. In the pathway’s “on” state, WNT ligand is recognized by FZ and LRP5/6, and LRP5/6 is phosphorylated. The WNT-FZ-LRP5/6 trimeric complex triggers the recognition of Dishevelled (DSH) and AXIN. β-catenin is not phosphorylated, translocates to the nucleus, and functions as a transcriptional coactivator to activate TCF/LEF family transcription factors. Prominent drug targets that aim to regulate WNT-responsive gene expression ^22^ include those that target (1) extracellular events, such as recognition of WNT by FZ and/or LRP5/6 (vantictumab and ipafricept), (2) cytoplasmic events, such as inhibition of DSH or stabilization of the AXIN/APC interaction (IWR-1; XAV939; 3289-8625; FJ9; NSC 668036; JW74), and (3) transcriptional activation, such as perturbing β-catenin function (PFK115-584; CGP049090; iCRT-3, -5, and -14; PRI-724). There are still other drugs that target events involved in WNT secretion to the extracellular space as well as other enzymes that regulate the pathway, but they are not shown in this schematic. Ub, ubiquitin. ( **B**) The sonic hedgehog (SHH) pathway is mediated by the receptors Smoothened (SMO) and Patched (PTC). In the pathway’s “off” state (in the event of low or no SHH ligand), SMO transport from intracellular vesicles to the membrane and its activity at the membrane are inhibited, in part by PTC. Members of transcription factor family GLI are inhibited by suppressor of fused (SUFU). Protein kinase A (PKA) phosphorylates the GLI transcription factors, which undergo proteasomal cleavage to yield a functional repressor form (GLI ^R^). GLI ^R^ translocates to the nucleus and inhibits target gene expression. In the pathway’s “on” state, SHH binds to and inhibits PTC and SUFU is inhibited. SMO levels at the membrane increase, leading to activation of GLI transcription factors, which translocate to the nucleus to activate SHH-responsive genes. Prominent drug targets that aim to regulate SHH-responsive gene expression ^13^ include those that target (1) extracellular events, such as SMO function, including by inhibition of SHH (purmorphamine, cyclopamine, vismodegib ^12^; sonidegib or Odomzo®, jervine; saridegib, CUR 61414, BMS-833923, glasdegib, PF-5274857, TAK-441, Taladegib, and SANT-1) and its binding to PTC (5E1, a monoclonal antibody), and (2) transcription activation, such as regulating GLI transcriptional activation (GANT61 and arsenic trioxide).

WNT medulloblastomas have evidence of WNT pathway ( Figure 2A) activation in their transcription profiles and almost uniformly have oncogenic mutations of *CTNNB1*, the proto-oncogene that encodes β-catenin. This subgroup comprises 10–15% of all medulloblastomas, found mostly in females aged 6–10. Their expression profiles map to multipotential progenitor cells of the lower rhombic lip ^15^. According to Phoenix *et al.* ^16^, there is a “signaling paradox” identified in which mutant catenin beta 1 (CTNNB1) protein drives constitutive, oncogenic WNT signaling in medulloblastoma ^17, 18^; this in turn silences normal WNT signaling in surrounding endothelial cells by producing inhibitors such as those of the secreted Frizzled-related protein (sFRP) family and WNT inhibitor factor 1 (WIF-1) that are secreted *in situ* ( Figure 2A). *CTNNB1* mutations are found in approximately 90% of WNT medulloblastomas, and nuclear accumulation of β-catenin is a biomarker for WNT pathway activation. WNT medulloblastomas form a highly hemorrhagic vasculature that lacks a blood–brain barrier ^16^. This may explain why these tumors are highly susceptible to chemotherapy, especially those that do not typically cross the blood–brain barrier, e.g. vincristine ^16^. Monosomy 6 is also found in 80–85% of WNT medulloblastomas (incidentally, these do not harbor telomerase reverse transcriptase [TERT] mutations), DEAD-box helicase 3 (DDX3X) mutations are found in 50% of WNT tumors, TERT mutations are found in 31% of WNT tumors ^19^, and the most common chromatin remodeling mutation found in WNT tumors is SMARCA4. Note that tumor suppressor protein p53 (TP53) mutations present in 15% of WNT tumors have no prognostic impact, unlike SHH TP53-mutant tumors that are associated with poor prognosis ^20^.

Patients with WNT medulloblastomas tend to have the most favorable outcomes; hence, current treatment protocols for WNT subgroup tumors are designed to minimize radiation and standard chemotherapy (refer to NCT01878617) and seek new treatments that target oncogenic mechanisms. Northcott *et al.* ^21^ suggested the use of panobinostat (a non-selective histone deacetylase inhibitor) (Novartis, USA) since the disruption of chromatin remodeling is thought to play a pivotal role in WNT medulloblastoma ^22, 23^. Anastas and Moon ^22^ also discuss a number of other potential inhibitors to the WNT pathway in their review.

## SHH subgroup of medulloblastoma

The SHH medulloblastoma group is a complex heterogeneous group of tumors, and the pathway is delineated in Figure 2B. SHH medulloblastomas have an intact blood–brain barrier and are less responsive to chemotherapy compared to WNT medulloblastomas ^16^. Infants (0–4 years) are more likely to have *SUFU* mutations than other age groups, and 42% of the infant samples have *PTCH1* alterations, while children (4–17 years) have a higher incidence of *MYCN* and *GLI2* amplifications, and 36% have *PTCH1* alterations ^24, 25^. Adults (anyone over the age of 17) are more likely to have *SMO* mutations than other age groups, and 54% of the adult samples have *PTCH1* alterations ^25^. Since there is a higher prevalence of *PTCH1* and *SMO* mutations in adult SHH medulloblastomas, this predicts responsiveness to inhibitors of the receptor SMO. Some tumors that arise from *SMO* mutations are sensitive to SMO inhibitors, but for others the *SMO* mutation renders the tumor insensitive ^24^. *MYCN* and *GLI2* amplifications or mutations also have been shown to be insensitive to SMO inhibitors ^12, 13^. SHH-inhibiting drugs that act downstream of SMO are currently in development ^24^. TERT promoter mutations are present in 38% of SHH medulloblastomas and, interestingly, are present in 80% of adult SHH tumors ^19^. Gorlin syndrome (also known as nevoid basal-cell carcinoma syndrome), caused by inherited germline *PTCH1* mutations or *de novo* (60% cases), is an autosomal dominant developmental disorder, and 5% of these individuals develop medulloblastomas during infancy ^26^. The outcome tends to be favorable as long as the patient does not have a *PTEN* or *GNAS* alteration ^26^. SHH-activated, *TP53*-mutant is a recent genetically defined WHO classification ^8^. *TP53* mutations occur in 13% of SHH tumors, and many of these are germline mutations (Li-Fraumeni syndrome) ^23, 24^. The SHH medulloblastomas with *TP53* mutations have extremely poor outcomes, and patients with these tumors should be selected for more intensive therapies and parents of those with germline mutations be offered genetic counseling ^14^. Protocols in development include removing DNA alkylating chemotherapy and minimizing radiation therapy in *TP53*-mutant tumors and relying instead on antimetabolite, microtubule-disrupting, or other types of chemotherapy ^25^. The SJMB12 study (NCT01878617) is currently prospectively evaluating treatment of SHH medulloblastoma in molecularly and clinically defined low, average, and high-risk patients and post-chemotherapy maintenance treatment with GDC0449 in children >12 years of age. Also, PI3K, mTOR, arsenic trioxide, and AKT inhibitors are potentially valuable in controlling specific targets in the SHH pathway and its interaction and links with the PI3K, mTOR, and AKT pathways ^25^.

## Non-WNT/non-SHH: group 3 medulloblastoma

Patients with group 3 medulloblastoma have a poor prognosis, and more than 50% of cases are metastatic at the time of diagnosis ^4^. Interestingly, older children with group 3 medulloblastomas have a 50% survival in 5 years if they have risk-adapted therapy. These tumors are more common in males and infants. This subgroup is notable for *MYC* over-expression, with *MYC* amplification observed in 17% of cases ^3^. Isochromosome 17q is a predictor of poor outcome in group 3 medulloblastomas ^27^. A large proportion of these group 3 medulloblastomas overexpress GABRA5, which may have therapeutic implications ^3, 28– 31^. Mutations in a number of genes involving chromatin remodeling affect 28.5% of group 3 tumors ^31^. Copy number changes that target genes in the transforming growth factor beta (TGF-β) signaling pathway affect approximately 20% of group 3 tumors ^31^. In addition, *PTV1* alterations are present in 12% of tumors, often as a fusion with *MYC* that drives its expression ^31^. The Wechsler-Reya group has shown HDAC and PI3K inhibitor combinations are promising in models of group 3 medulloblastoma ^30^. The SJMB12 study (for all medulloblastoma subgroups, mentioned in the previous section) is also prospectively evaluating the use of pemetrexed, gemcitabine, vincristine, cisplatin, and cyclophosphamide in the high-risk medulloblastoma cases, and this study is currently open.

## Non-WNT/non-SHH: group 4 medulloblastoma

Group 4 medulloblastoma is also known as the glutamatergic subgroup, and it is the commonest molecular subgroup. For specific characteristics of this subgroup, please refer to Table 1. The average-risk patients in this subgroup have excellent survival with the current standard-of-care treatment options ^11^. It has a phototransduction and neuronal signature in its transcription profile, as initially described by Cho *et al.* ^3^. However, more recently, the homeobox transcription factor Lmx1A has been identified as a master regulator transcription factor of group 4 medulloblastomas ^32^. Lmx1A is important in the normal development of cells in the upper rhombic lip and cerebellum, and it is also critical for the development of midbrain dopaminergic neurons ^31, 32^, which are thought to be where group 4 tumors originate. Interestingly, this subgroup of medulloblastoma is three times more common in males than in females ^4^. More recently, the presence of metastatic disease at diagnosis or chromosome 11 loss and chromosome 17 gain appear to dictate the prognosis in this subgroup of medulloblastoma patients ^27^. In addition, copy number changes in target genes that are important in the nuclear factor kappa-light-chain-enhancer of activated B cells (NF-κβ) signaling pathway are found in this subgroup ^29, 31^. The most common chromosomal aberration found in group 4 tumors is isochromosome 17q; it is also found to a lesser degree in group 3 tumors ^4^.

## Recurrent medulloblastomas

Despite the subgroup designation of medulloblastomas, at recurrence (tumor relapse with or without leptomeningeal dissemination), there is substantial divergence of the dominant clone ^33^. Interestingly, however, subgroup classification is maintained at recurrence or metastasis ^33^. Most medulloblastomas that recur post-cytotoxic therapies are fatal, and the patterns of relapse tend to be subgroup specific. The concept of intratumor heterogeneity was initially described by Gerlinger *et al.* ^34^ for renal cell cancer, and more recently for medulloblastoma ^35^. In addition, the microenvironment of the tumor will have implications for drug design, including immunotherapies ^36^. This might be why, despite the design of targeted clinical trials, there is failure of these targeted agents ^37^.

## Secondary tumors following treatment of medulloblastomas

There is the added caveat of medulloblastoma patients who survive radiation and chemotherapy who then go on to develop other tumors such as meningiomas and gliomas ^37^, etc. Packer *et al.* ^37^ in the Children’s Oncology Group A9961 trial described a cohort of 379 patients (aged 3 to 21), and 15 of these patients went on to develop secondary tumors 5.8 years after their initial diagnosis of medulloblastoma. Thus, recurrence and secondary cancers post-standard treatment of medulloblastoma patients makes monitoring of this pediatric population well into adulthood a necessity.

Many groups are looking at targeted next-generation sequencing approaches in neuro-oncology in the initial diagnosis and recurrent setting to improve treatment options ^38^, since there is a lack of validated targets for non-SHH/WNT medulloblastoma that sequencing may help unravel. In addition, many cancers, including medulloblastomas, have DDX3X mutations, as briefly discussed in the WNT section, and recent work has shown that mutations in this gene result in global reduced translation ^39^. This may confer certain survival advantages, perhaps in certain microenvironments, which may aid in designing alternative therapeutic options ^36^.

## Future directions

Despite dissecting medulloblastomas in the “genomic sense”, it is clear that therapy cannot be dictated by the subgroups alone. There are other key players, and epigenetics has a big role to play ^40^. Indeed, it is becoming increasingly clear that global changes in the epigenetic architecture are signatures of cancer and tumorigenesis. It was described in 2014 by Diede *et al.* ^41^ that DNA methylation probably prevents normal differentiation in pediatric cancers. It is known that focal regions of low methylation linked to transcription-factor-binding sites shed light on differential transcriptional networks between subgroups; however, increased methylation correlates with gene expression ^41, 42^.

Although in its infancy in medulloblastoma, proteomics is another strategy being utilized to analyze the tumor microenvironment, since other metabolites, such as small peptides and lipids, can be crucial in regulating tumor development ^43^. Summarizing the future of medulloblastoma treatment, numerous strategies for designing and tailoring treatment for medulloblastoma will evolve to harness the different technologies, such as genomics, methylomics, and proteomics. Even though personalized medicine is not de rigueur in medulloblastoma management, it is something that is on the horizon.

## Abbreviations

CTNNB1, catenin beta 1; DDX3X, DEAD-box helicase 3; SHH, sonic hedgehog signaling pathway; TERT, telomerase reverse transcriptase; TP53, tumor suppressor protein p53; WNT, Wnt signaling pathway.
